# Effect of Pd/Pt decoration on MoSSe monolayer for CH_4_ signature through surface adsorption mechanism

**DOI:** 10.1038/s41598-023-49028-x

**Published:** 2023-12-12

**Authors:** Bindiya Babariya, Sanjeev K. Gupta, P. N. Gajjar

**Affiliations:** 1 Department of Physics, University of School of Sciences, Gujarat University, 380 009, Ahmedabad, Gujarat India; 2 Computational Materials and Nanoscience Group, Department of Physics, St. Xavier’s College, Ahmedabad, 380009 India

**Keywords:** Electronic and spintronic devices, Sensors

## Abstract

Considering the current breakthrough in gas sensor technology, we have examined impact of CH_4_ in the vicinity of pristine MoSSe and Pd/Pt decorated MoSSe monolayers using first principles approach. The negative formation energies confirm structural stability of considered monolayers. The pristine MoSSe monolayer is semiconductor having 1.52 eV direct band gap. This value decreases in the presence of Pd/Pt adatom. Further, adsorption strength of CH_4_ to monolayers is validated by sensing parameters such as adsorption energy, recovery time, charge transfer and work function. Though we found maximum adsorption energies of − 0.674 and − 0.636 eV for adsorption on Se site of Pd/Pt decorated MoSSe monolayers, the overall sensing response also reveals high sensitivity for Se surface. However, both sites S and Se are favorable for CH_4_ adsorption. When CH_4_ is activated on Pd/Pt decorated monolayers, band gaps vary with marginal alterations and transform to direct type. Moreover, optical dielectric response alters strongly in the visible region after activation of CH_4_ on to Pd/Pt decorated MoSSe monolayers. This result identifies sensitivity response in the presence of methane which may detect CH_4_ gas easily in visible region. Generally, these interesting results of methane sensing study provoke Pd/Pt decorated MoSSe monolayers to be good sensing nano-device.

## Introduction

The exponential growth of designing extraordinary nano-dimensional integrated electronic and optoelectronic devices leads to unbeatable progress in the commercial arena. With progress in sensing technology over the past decade, researchers have focused mainly on toxic gas sensing mechanisms, especially N and C based toxic gases such as NO, NO_2_, CO and CO_2_ on 2D nanomaterials. Specifically, transition metal dichalcogenides (TMDs) based toxic gas sensors have been studied more efficiently as they exhibit ultrahigh surface dimensions, specific chemical reactivity and intriguing sensing properties with better sensitivity and selectivity^1–8^. In the last few years, NO_2_ and NH_3_ exposition upon MoSSe monolayer reveal chemical interactions with fascinating sensing properties for Se site^2,3^ and depict that strain and electric field modulation enhance capturing performance. Moreover, Cui et al.^4^ pointed out that NO_2_ sensing upon HfSe_2_ monolayer can be functionalized by Pd doping and indicate recycle use as resistance type gas sensor with much higher adsorption energy (~ eight times) compared to bare HfSe_2_ monolayer. When exposed to CO, CO_2_, NO, NO_2_, H_2_S, and SO_2_ gas molecules, NbSeTe monolayer shows distinct transport properties along with sensing behavior. These variations expose 98% and 97% of sensitivity for NO and SO_2_ gases, respectively on Te side with noticeable electron transmission^5^. Furthermore, Wu et al.^6^ synthesized WSe_2_ monolayer which exhibits fast recovery, reproducibility and selectivity especially upon NO_2_ detection up to 5 ppm concentration level at 250 °C in comparison with other gases (CO, CO_2_, H_2_ and NH_3_) detection. However, it has also been interesting to detect other abundant gases such as C_2_H_2_, C_2_H_4_, CH_4_, etc. through various 2D nanolayers based sensing devices.

The most abundant methane gas has played a major role in greenhouse effect after CO_2_ in the atmosphere. The rapidly growing methane concentration since 2014^9^ is very dangerous to human health. It makes humans feel suffocative at extent concentration and risky to skin^10^. Apart from this, odorless and flammable characteristics of methane at room temperature may cause high explosive risk while gas leakage^11^. Methane is also advantageous for industrial and environmental practical applications. Thus, detection of coalmine methane has attracted significantly due to dominant effect on global warming and mankind safety. This sensing of methane desires reliable and sensitive 2D materials as adsorbate because of more stability of C–H bond in non-polar CH_4_ molecule^12^.

So far, many researchers have reported the adsorption of domestic gas methane onto 2D nanolayers system^13–29^. Recently, Chen et al.^13^ studied sensing mechanism of C_2_H_4_, CH_4_, H_2_ and H_2_O on CuO and NiO metal oxide doped SnS_2_ monolayers. Amongst, they proposed enhanced conductivity (from electron transfer) with fast desorption process after methane physisorption on NiO metal oxide doped SnS_2_ surface. As a possible approach to reach excellent methane sensing, Hussain et al.^14^ analyzed C and Ga doped ZnO nanosheets which exhibit 6.1 and 7.5 times stronger binding of CH_4_ adsorption, respectively, promoting it as an effective nano sensor. Also, Wang et al.^15^ synthesized the Vo-rich mesoporous ZnO nanosheet which improves UV-activated methane gas sensing performance at room temperature. Meantime, many studies have been carried out by substituting or decorating 2D monolayers through foreign atom to enhance sensitivity of gas sensor. For instance, Yu et al.^16^ showed Pd-C_3_N nanosheet as a potential candidate for CH_4_ sensing with higher affinity compared to pristine C_3_N monolayer. However, Pd/Pd_2_ decorated graphene induces chemical interaction upon activation of methane gas with strongest adsorption energy of − 1.43/− 2.27 eV, respectively^17^. Also, Pt/Al doped graphene exhibit better sensing results with good adsorption strength with respect to isolated monolayer^18,19^. Yang et al.^20^ reported effects of various types of graphene stacking on methane adsorption performance showing highest adsorption energy for Z-AB graphene and revealed that A-order graphene would be better for methane sensing after foreign atom doping. Correspondingly, Lam and co-workers^21^ fabricated GO and RGO-SnO_2_ heterostructure and reported sensing mechanism towards the CH_4_ by incorporating with various reducing agents.

In this context, Ti doped γ-graphyne demonstrated significant binding energy of − 4.96 eV, indicating chemisorption of methane on the same monolayer with fast reversibility of 9.7 ms^12^. Also, Abbasi^22^ examined the adsorption of CH_2_O and CH_4_ on stanene monolayer and encouraged favorable and considerable changes in the electronic properties. Wang et al.^24^ reported enriched conductivity along with improved sensitivity and recovery time for GeSe monolayer with Pd/Pt modification. In contrast, CH_4_ adsorption on SnO_2_–GeSe monolayer decreases conductivity^25^. Very recently, Pt-decoration on WS_2_ monolayer increases the adsorption energy up to 1.2 eV and enhances sensitivity up to 22%^26^. Fe decorated blue phosphorene also exhibits reduced band gap by two-third proportion and magnetic moment by 0.23 µB after activation of CH_4_ on surface^27^. Apart from these tailored monolayers, prominent 2D TMDs were also proposed to be capable for capturing toxic and fuel gases at room temperature. With this regard, Santos and Putungan^28^ reported methane adsorption toward strained 1T′-MoS_2_ monolayer which improves MoS_2_–CH_4_ interaction in contrast to pristine monolayer. Moreover, an effect of atomic scale defects in MoX_2_ (X = S, Se, Te) monolayer modulated adsorption strength and demonstrated best adsorption for CH_4_ in case of divacant (chalcogen vacancy) MoTe_2_^29^.

With this consideration for detecting CH_4_ significantly, we have selected both S and Se sites of MoSSe monolayer for adsorption. To enhance sensing performance, we have also modified MoSSe monolayer by adding Pd/Pt atom on both S and Se sites. We have investigated various structural orientations to check favorable adsorption sites. The binding strength of interaction between monolayer and CH_4_ has been studied within frame of adsorption energy, recovery time, charge transfer and work function. The results indicate higher binding strength of CH_4_ in the vicinity of Se site of Pd/Pt decorated MoSSe monolayer. Furthermore, for in depth detail toward optical sensing, we have explored dielectric function under the effect of CH_4_ for all monolayers. The overall findings manifest S and Se both surfaces to be better selective for CH_4_ gas sensing.

## Computational approach

For all ab-initio calculations of methane gas sensing onto both sites S and Se of Janus MoSSe monolayer, SIESTA (Spanish Initiative for Electronic Simulations with Thousands of Atoms) simulation code^30^ based on density functional theory (DFT) has been employed. The first principles calculations rely on Perdew–Burke–Ernzerhof (PBE) pseudopotential within generalized gradient approximation (GGA)^31^ exchange and correlation functional to treat electron–ion interaction. We have also used van der Waals density functional (vdW-DF) proposed by Dion et al.^32^ to counter interaction between adsorbate and gas molecule. A vacuum of 16 Å is considered to prevent adjacent sites influence. The double-ζ polarized (DZP) basis sets along with cut-off energy of 400 Ry have been considered. Moreover, 15 × 15 × 1 and 25 × 25 × 1k-point Monkhorst–Pack sampling grid^33^ in the Brillouin zone are used for structural optimization and sensing properties, respectively. The energy shift is set to 0.02 Ry for self-consistent field convergence. The Hellmann–Feynman forces up to 0.01 eV/Å is applied on each atom using the conjugate gradient (CG) algorithm. At first, we focused on stability of pristine MoSSe monolayer which is reported in previous study^34^. Also, we have confirmed stability of Pd/Pt decorated MoSSe monolayers in terms of binding strength using formation energy (*E*_f_) by the following relation where, $E_{{{\text{MoSSe}} + {\text{Pd}}/{\text{Pt}}}}$, $E_{{{\text{MoSSe}}}}$ and $E_{{{\text{Pd}}/{\text{Pt}}}}$ stand for total computed energy of modified monolayer, pristine monolayer and isolated Pd/Pt atom, respectively.

1 $$E_{{\text{f}}} = E_{{{\text{MoSSe}} + {\text{Pd}}/{\text{Pt}}}} - E_{{{\text{MoSSe}}}} - E_{{{\text{Pd}}/{\text{Pt}}}}$$

In addition, spin polarization calculations are also performed to verify spin dependent behavior of Pd/Pt doped systems in electronic properties. To figure out adsorption interaction strength of CH_4_ molecule with MoSSe monolayer, we have determined adsorption energy ($E_{{{\text{ads}}}} )$ using the formula:

2 $$E_{{{\text{ads}}}} = E_{{{\text{monolayer}} + {\text{CH}}_{4} }} - \left( {E_{{{\text{monolayer}}}} + E_{{{\text{CH}}_{4} }} } \right)$$

where $E_{{{\text{monolayer}} + {\text{CH}}_{4} }}$, $E_{{{\text{monolayer}}}}$ and $E_{{{\text{CH}}_{4} }}$ represent total obtained energy of MoSSe monolayers in the presence of methane, host monolayers (respective pristine or Pd/Pt decorated) and isolated methane molecule, respectively. The Charge transfer has been determined by means of Hirshfeld analysis as incorporated in siesta.

Further, to emphasis impact of CH_4_ adsorption on dielectric constant, we have used method of random phase approximation (RPA). The dielectric function is stated as $\varepsilon \left( \omega \right) = \varepsilon^{\prime}\left( \omega \right) + i\varepsilon^{\prime\prime}\left( \omega \right)$. Here, $\varepsilon^{\prime\prime}\left( \omega \right)$ and $\varepsilon^{\prime}\left( \omega \right)$ are imaginary and real part of dielectric function, respectively which are examined using the Kramers–Kronig transformation^35^.

## Results and discussion

### Structural and electronic properties

**Figure 1:**
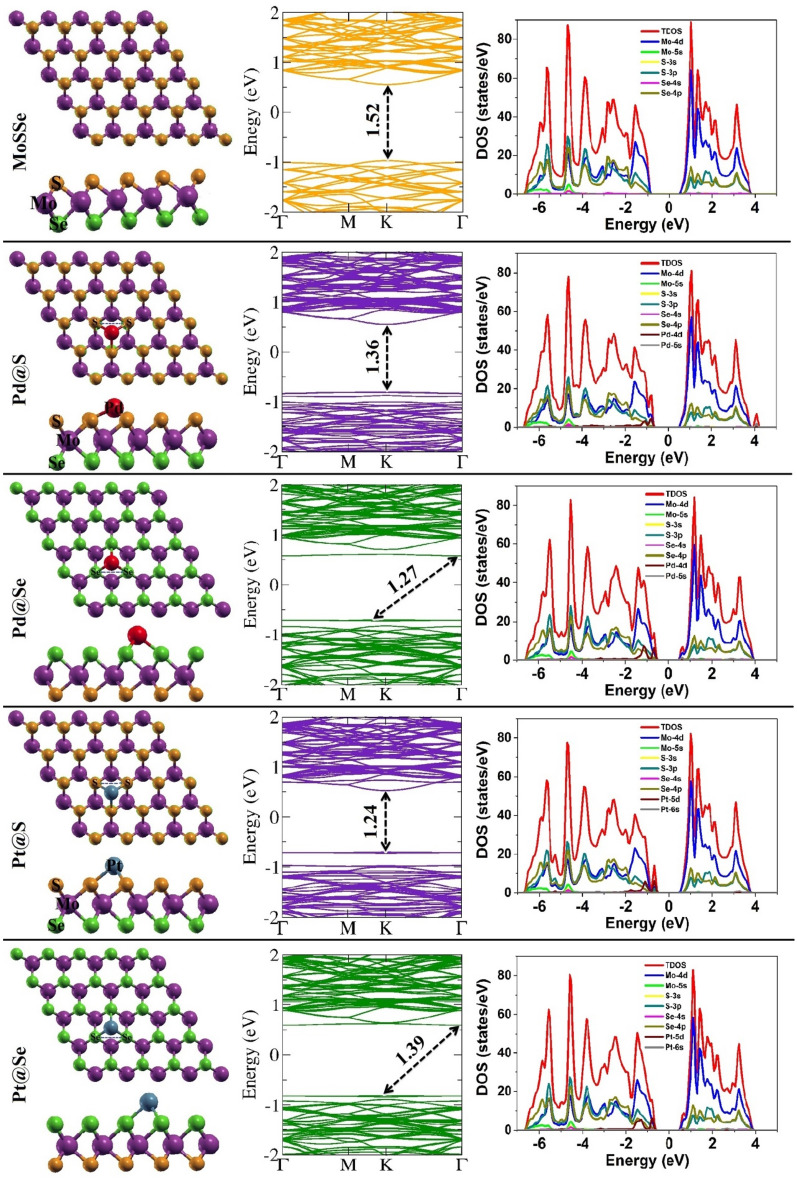
The schematic presentation of side and top views of Janus MoSSe monolayers along with Pd/Pt decoration on favorable S and Se sites, corresponding electronic band structure and DOS.

The 2H phase of Janus TMDs is the most stable among all possible phases such as 1T, 1T′ and 2H^36–38^. This 2H phase of Janus MoSSe monolayer is structured through sandwiched transition metal Mo between two different chalcogen atoms S and Se. We considered 5 × 5 × 1 supercell of MoSSe monolayer to investigate structural and sensing parameters. The relaxed geometry of pristine MoSSe monolayer reveals minimal lattice constant of 3.28 Å^34^ while, lattice constant varies a bit (in meV) for Pd and Pt decorated MoSSe monolayers as mentioned in Table 1. These side and top views of optimized geometries after Pd/Pt modification on to MoSSe monolayer are shown in Fig. 1. Here, we selected either side of pristine MoSSe monolayer for doping (*i.e.*, Pd@S and Pd@Se denote Pd modification on to S and Se site, respectively, likewise for Pt). It is seen that Pd/Pt atom occupied steady position over the hollow space on both sites S and Se of MoSSe monolayer. These Pd and Pt atoms on to MoSSe sheet are settled at the distance of 0.88/1.51 Å and 1.48/1.75 Å on S/Se sites, respectively. Besides, these foreign atoms are attached to the sheet through chemical bonding. The geometry of nearest attached atoms to doping area is influenced (as highlighted in Fig. 1) because of Pd/Pt modification as S–S atom separation changes significantly by 0.46 Å due to Pd decoration on S site which stretches neighbor atoms, while Pd atom deposition on Se site does not change Se–Se distance (0.06 Å) rigorously. Likewise, Pt decoration on S/Se sites results in increased S–S/Se–Se separation by 0.11/0.03 Å. Correspondingly, Mo–S/Se bond lengths increase in the range of 0.02–0.08 Å after Pd/Pt doping on MoSSe monolayer.

**Table 1:** The optimized lattice constant, bond distance (d) of Pd/Pt to sheet atom S/Se, formation energy (*E*_f_), energy gap (*E*_g_), work function (Φ) and charge transfer (ΔQ).

System	Lattice constant (Å)	d (Å)	*E*_f_ (eV)	*E*_g_ (eV)	Φ (eV)	ΔQ (e)
MoSSe@S	3.28	–	− 3.86^34^	1.52	3.58	–
MoSSe@Se					3.59	
Pd@S	3.273	0.88	− 5.15	1.36	3.94	0.129
Pd@Se	3.268	1.51	− 4.56	1.27	4.06	0.103
Pt@S	3.267	1.48	− 4.10	1.24	3.89	0.375
Pt@Se	3.267	1.75	− 3.82	1.39	4.00	0.351

After geometry optimization, firstly we focused on structural stability of Pd/Pt modified monolayers through formation energy as tabulated in Table 1. Usually, more negative formation energy, more stability of monolayer. Here, we found maximum formation energy of − 5.15 eV for Pd decorated MoSSe monolayer, indicating higher stability with respect to Pt modified monolayers. The electronic band structure along with orbital contribution in the configuration of total and projected density of states (DOS) have also been displayed in Fig. 1. The obtained band gap values confirm semiconductor character of MoSSe monolayers before and after Pd/Pt decoration. The direct band gap of 1.52 eV for pristine MoSSe monolayer is in accordance with deviation of ~ 2.6% to earlier reported value 1.56 eV^39^. Pd/Pt medication on S site of MoSSe monolayers introduce new impurity bands near valence band maxima (VBM) which decrease direct band gap as 1.36/1.24 eV at K-point, is in coordination to bands of pristine monolayer. While impurity bands in both regions i.e., near VBM and CBM (conduction band minima) are seen due to Pd/Pt modification over Se-site. These results in transition from direct to indirect band gap of 1.27/1.39 eV along Γ → K point. This decrease in band gap of monolayers after Pd/Pt decoration (with PBE functional) also agrees with recently reported results^26,40,41^. Also, the effect of included spin orbit coupling (SOC) and spin polarizations on the electronic band structures and DOS of pristine and doped MoSSe monolayers is very negligible. Therefore, these results are shown in Figs. S1 and S2, respectively. However, this underestimated band gap for pristine and modified monolayers can be enhanced by applying hybrid or self-correction to functionals^42^. Correspondingly, the Fermi level slightly shifts towards conduction band and valence band after Pd/Pt modification on S and Se sites of MoSSe monolayers, respectively. These impurity bands are almost flat where major dense contribution is due to *Pd*-4*d* and *Pt*-5*d* orbitals near VBM and CBM. For pristine and Pd/Pt decorated monolayers, the main orbital contribution in conduction bands is from *Mo*-4*d* orbitals which reveals stronger delocalization of 4*d* electrons. This main peak in electron density distribution lies at about 1.1 eV in the conduction band. Apart from *Mo*-4*d* orbitals, *S*-3*p* and *Se*-4*p* orbitals also play vital roles in valence bands. Moreover, *s* orbitals of S, Se and Pd/Pt contribute to occupied energy states far from the Fermi level.

Further, we figured out modulation in work function and charge transfer value due to Pd/Pt decoration on to MoSSe monolayer as mentioned in Table 1. The significant changes within range of 0.3–0.4 eV are noticed in work function after doping with respect to Φ of pristine MoSSe monolayer. This shows a major change in withdrawal energy required for electrons to escape from surface due to Pd/Pt decoration. This result also matches well with electrostatic potential height as encircled in Fig. S3, where infinitesimal variation is remarked at the end of the barrier height. However, significant charges are transferred from Pd/Pt to MoSSe sheet means MoSSe act as an acceptor. This noteworthy charge transfer indicates strong interaction between dopant and monolayer, exhibiting higher binding strength which is also manifested from connection of dopant Pd/Pt to the monolayers through chemical bond.

### Sensing properties

**Figure 2:**
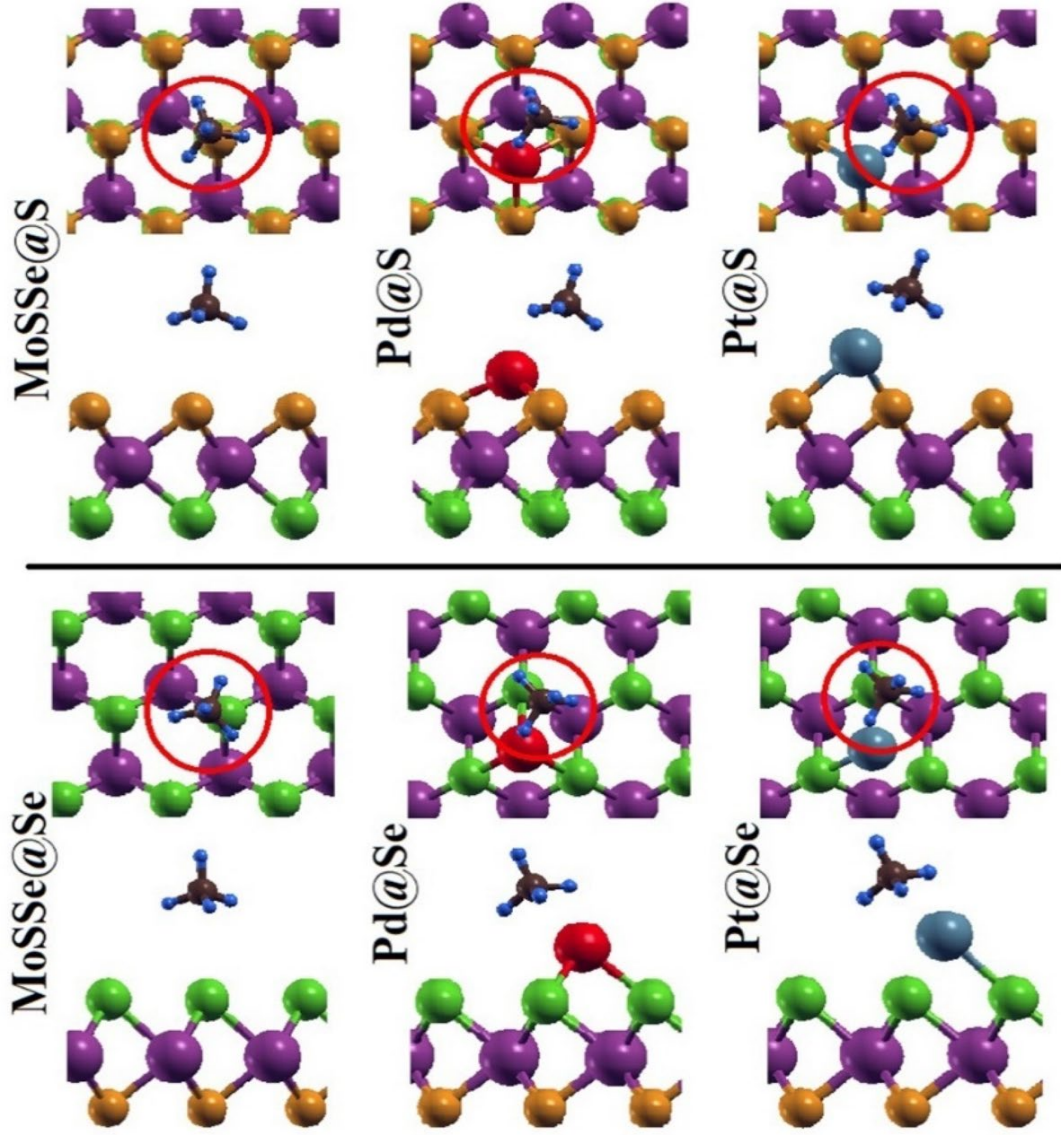
The top and side views of optimized geometry of pristine and Pd/Pt decorated MoSSe monolayers after CH_4_ adsorption.

To explore the sensing nature of CH_4_, we have again considered both sites S and Se for an adsorption on to pristine MoSSe and respective Pd/Pt decorated monolayers. We have adopted two different orientations for CH_4_ adsorption (1) top hydrogen of C-H bond in tetrahedral shape is being closure toward monolayer and (2) top hydrogen is away from the monolayer i.e., bottom three hydrogen atoms are being closure to MoSSe monolayer. Also, we have chosen two different positions on to monolayer i.e., upon hollow space of hexagon and Mo-S/Se bond. Amongst all, we have marked optimized CH_4_ on to MoSSe monolayer with feasible binding orientation at lowest energy in Fig. 2. From optimization, it is noticed that despite of different orientations, CH_4_ is being tilted toward adatom Pd/Pt while adsorption upon Pd/Pt modified monolayers in both cases. Also, CH_4_ has occupied the final position above Mo–S/Se bonds for all monolayers. Here, among all adsorption configurations, noticeable change in bond length is only found for Pt@Se system after CH_4_ adsorption. Probably, absence of one Pt-Se bond is either due to relatively lower formation energy of Pt with Se atom among all structures (as mentioned in Table 1) or owing to interaction taking place between *d* and *f* orbitals of Pt atom and *p* orbital of Se atom in the presence of methane molecule. This can also be visualized in DOS as density of Pt*-5d* and Se*-4p* orbitals relatively decrease at energy nearly about 0.8 eV. Moreover, C–H bond length is 1.10 Å and the bond angle of H–C–H is about 109.77° for methane molecule. The corresponding adsorption equilibrium height (h) of CH_4_ gas molecule from S and Se sites of MoSSe monolayers is compiled in Table 2. It is found that equilibrium height from H atom to nearest in-plane atom on Se site of monolayers toward CH_4_ is short compared to same on S site. However, chemical bonding between adsorbent and adsorbate is missing for all monolayers indicating physical adsorption of methane on to MoSSe monolayer. Besides, Pd/Pt atom to S and Se bond length is increased by 0.04/0.02 Å and 0.07/0.02 Å, respectively in the presence of CH_4_ molecule.

**Table 2:** The examined sensing parameters, adsorption energy (E_ads_), equilibrium height (h), energy gap (E_g_), recovery time (τ), work function (Φ) and charge transfer (ΔQ) for methane adsorbed MoSSe monolayers.

System	E_ads_ (eV)	h (Å)	E_g_ (eV)	τ (s)	Φ (eV)	ΔQ (e)
MoSSe@S	− 0.274	2.95	1.52	40.3 × 10^–9^	4.00	− 0.032
MoSSe@Se	− 0.269	3.07	1.52	32.7 × 10^–9^	3.91	− 0.035
Pd@S	− 0.446	2.02	1.36	31.3 × 10^–6^	3.92	− 0.029
Pd@Se	− 0.674	1.17	1.29	215 × 10^–3^	3.95	− 0.034
Pt@S	− 0.553	1.94	1.14	1.96 × 10^–3^	3.87	− 0.047
Pt@Se	− 0.636	1.12	1.42	48.6 × 10^–3^	3.93	− 0.035

Next, we begin with adsorption response of pristine and Pd/Pt decorated MoSSe monolayers toward CH_4_ gas molecule which is examined in respect of sensing parameters like adsorption energy, recovery time, charge transfer and work function as formatted in Table 2. Generally, the prior factor to understand gas sensing nature of monolayer as chemisorption or physisorption is adsorption energy. Here, the calculated adsorption energy for selected adsorption sites of MoSSe monolayer is negative, suggesting exothermic adsorption process in the vicinity of CH_4_ molecule. The adsorption energy for MoSSe monolayer toward CH_4_ is increased significantly (by ~ 0.3 eV) which affirms improved adsorption ability in the presence of Pd/Pt atom. Such more negative energy concludes more binding stability along with strong interaction for Pd/Pt decorated MoSSe monolayers as methane sensor. The maximum negative energy of about − 0.6 eV is obtained for CH_4_ adsorption on Se site of Pd/Pt decorated monolayers. Nevertheless, this extreme adsorption energy is also highest in earlier reported data. In detail, Pd^17^ and Pt-decorated^18^ graphene (of same structural configuration as TMDs) exhibits energy of − 0.45 and − 0.485 eV, respectively toward methane physisorption.

Another important index to look over better sensing is recovery time i.e., the time required in desorption process of gas molecules from the surface of sensing device. It is challenging for any gas sensor to desorb gas molecules from the monolayer surface within a short time for recycle use. Desorption will be more difficult if the interaction between surface and adsorbed molecule i.e., binding strength is too strong. Here, the recovery time (τ) is estimated from the adsorption energy using transition state theory with Van’t-Hoff-Arrhenius expression^43,44^ as follow:

3 $$\tau = \nu^{ - 1} {\text{exp}}\left( { - \frac{{E_{ads} }}{{K_{B} T}}} \right)$$

where T is temperature, *K*_*B*_ denotes Boltzmann’s constant considered to be 8.62 × 10^−5^ eV/K and ν is attempt frequency factor. In visible region, it is considered to be 10^12^ s^−1^ at temperature 300 K^43,45^. The results show that only Pd decorated monolayer (Se site) requires 215 ms for desorption process. All other results depict fastest desorption process of methane within time of the order of ns to ms for both pristine and Pd/Pt decorated MoSSe monolayers. Such characteristics shed light on potential of MoSSe for reusability as a methane gas sensor at lower temperature (300 K).

One more essential index to judge sensing performance in detail is work function (Φ). The work function is related with maximum energy required for electron to escape from the monolayer surface to vacuum. It is remarked that calculated work function is somewhat sensitive in the presence of CH_4_. Consequently, marginal alterations of 0.01–0.02 eV are observed for CH_4_ adsorbed S-site of Pd/Pt decorated monolayers with respect to pristine monolayer. Moreover, large variation of 0.3–0.4 eV for pristine MoSSe and moderate variations of 0.11 and 0.07 eV in case of Se sites of Pd/Pt@MoSSe monolayers are noticed due to CH_4_ adsorption, respectively. This may change conductivity due to chemical interaction undergoing between adsorbate and adsorbent which affect surface dipole at interface. The corresponding electrostatic potential profile also varies due to gas adsorption. Figure S4 displays the planer average of electrostatic potential along the z direction. It clearly shows the influence of methane adsorption at the edge of the reference level for either sites S and Se of pristine and Pd/Pt decorated MoSSe monolayers. Comparing the work function for S site to Se site toward methane adsorption, work function for Se site reveals major variation from MoSSe to Pt-decorated monolayers. This apparently justifies through remarkable shift to higher distance observed for S site in contrast to Se site in the presence of CH_4_. Besides, extended bottom difference of potential reveals electronegativity difference between S and Se. In the preceding sensing parameters, we figured out charge transfer between gas and monolayers after CH_4_ adsorption. The negative values of charge transfer confirm that it completely alters from monolayer to gas transfer. Hence, CH_4_ behaves as acceptor and MoSSe monolayers act as donor. Also, remarkably less charges are transferred from monolayers to gas compared to that from dopant to monolayer.

### Influence of CH_4_ adsorption on electronic properties

**Figure 3:**
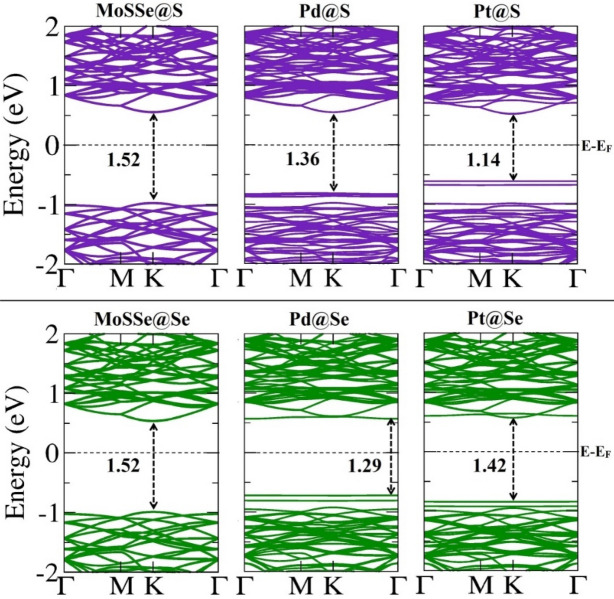
Electronic band structures for pristine and Pd/Pt decorated MoSSe monolayers after CH_4_ adsorption. The Fermi level is set to zero value. Band gap shown is in eV.

**Figure 4:**
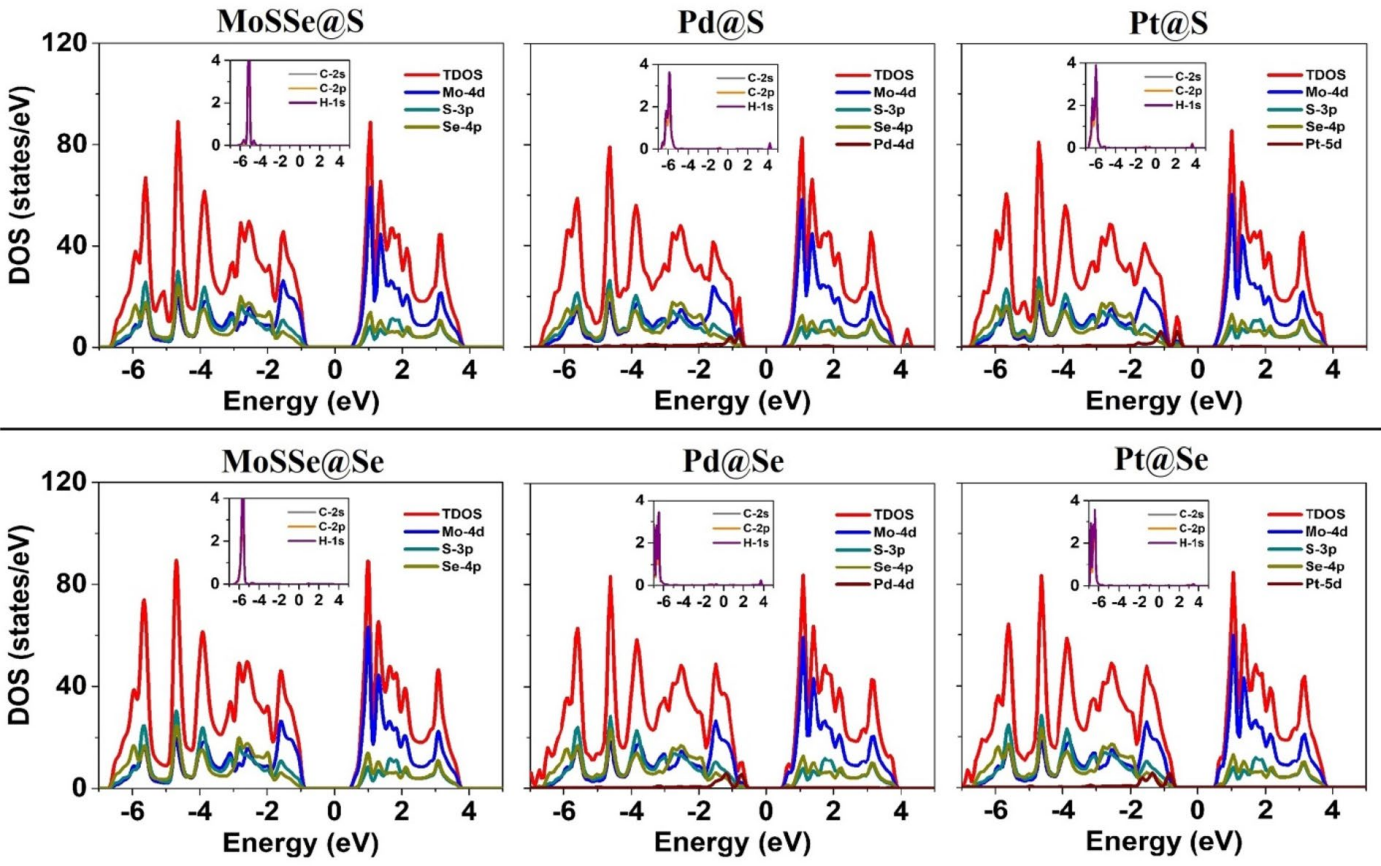
Total and projected density of states of pristine and Pd/Pt decorated MoSSe monolayers after CH_4_ adsorption.

Following the discussion, we have explored electronic properties as electronic band structure and DOS in the presence of CH_4_ molecule as illustrated in Figs. 3 and 4, respectively. The electronic band structures correspond to pristine and Pd/Pt decorated MoSSe monolayers exhibit semiconducting character with direct band gap, ranging from 1.14 to 1.52 eV. Also, in band structures, VBM and CBM for all monolayers are located mostly at K point. It is clearly seen that the band gap for pristine monolayer remains same before and after CH_4_ adsorption for both sites. Besides, band gap of 1.36 eV for adsorption on Pd@S monolayer does not change in the presence of CH_4_, while in the same proximity, band gap is increased by 0.02 eV for Se-site. Apparently, it is observed that Fermi level shifts toward conduction band in the presence of CH_4_ on S and Se sites of monolayers and correspondingly, impurity states also move to the conduction band. Thus, CH_4_ influences the band gap of Pt decorated monolayers as it decreases by 0.10 eV and increases by 0.03 eV due to adsorption on S and Se sites, respectively. However, these impurity bands, appeared due to Pd/Pt modification on monolayers witness flat as of before adsorption. Consequently, this change in band gap regulates the conductivity according to the relation $\sigma \propto {\text{exp}}\left( { - \frac{{E_{g} }}{{2k_{B} T}}} \right)$, where *E*_*g*_ refers to band gap, *k*_*B*_ is Boltzmann constant and T is temperature.

Meanwhile, electronic DOS and PDOS have been examined to understand orbitals interaction of monolayers and CH_4_ where individual contribution from each orbital is presented within energy span of − 7 to 5 eV in Fig. 4. The orbital states from CH_4_ after interacting with monolayers have been displayed separately. It is evidently seen that tendency of TDOS is congruent for all monolayers in the affinity of CH_4_ molecule except near the Fermi level. The second alike witness after adsorption is the major contribution from *Mo*-4*d* orbital near CBM and VBM. Also, *p* orbitals of chalcogen atoms S and Se mainly contribute to valence bands and states located far from the Fermi level are mainly due to *s* orbitals of S/Se and Pd/Pt atoms. Besides, no specific variation except shifting in Fermi energy and impurity bands are observed near CBM and VBM due to CH_4_ adsorption. Also, almost identical states as before adsorption are seen near Fermi level due to *Pd*-4*d* and *Pt*-5*d* orbitals. However, strong peak due to *C*-2*p* and *H*-1*s* orbitals is seen in valence bands (at ~ − 6 eV). This peak shifts little away (~ 1 eV) from the Fermi energy for Pd/Pt decorated monolayers with respect to that observed in pristine monolayer after CH_4_ adsorption. Commonly, σ → σ* transition takes place during band formation in saturated hydrocarbons like methane. Though, when CH_4_ comes in contact with pristine and Pd/Pt decorated MoSSe monolayers, n → σ* transition may also arise due to physical interaction of CH_4_ with monolayers. Moreover, hybridization between *d* and *p* orbitals occurs due to overlapping of 4*d* and 3*p*–4*p* orbitals in valence bands and conduction bands.

### Optical properties

**Figure 5:**
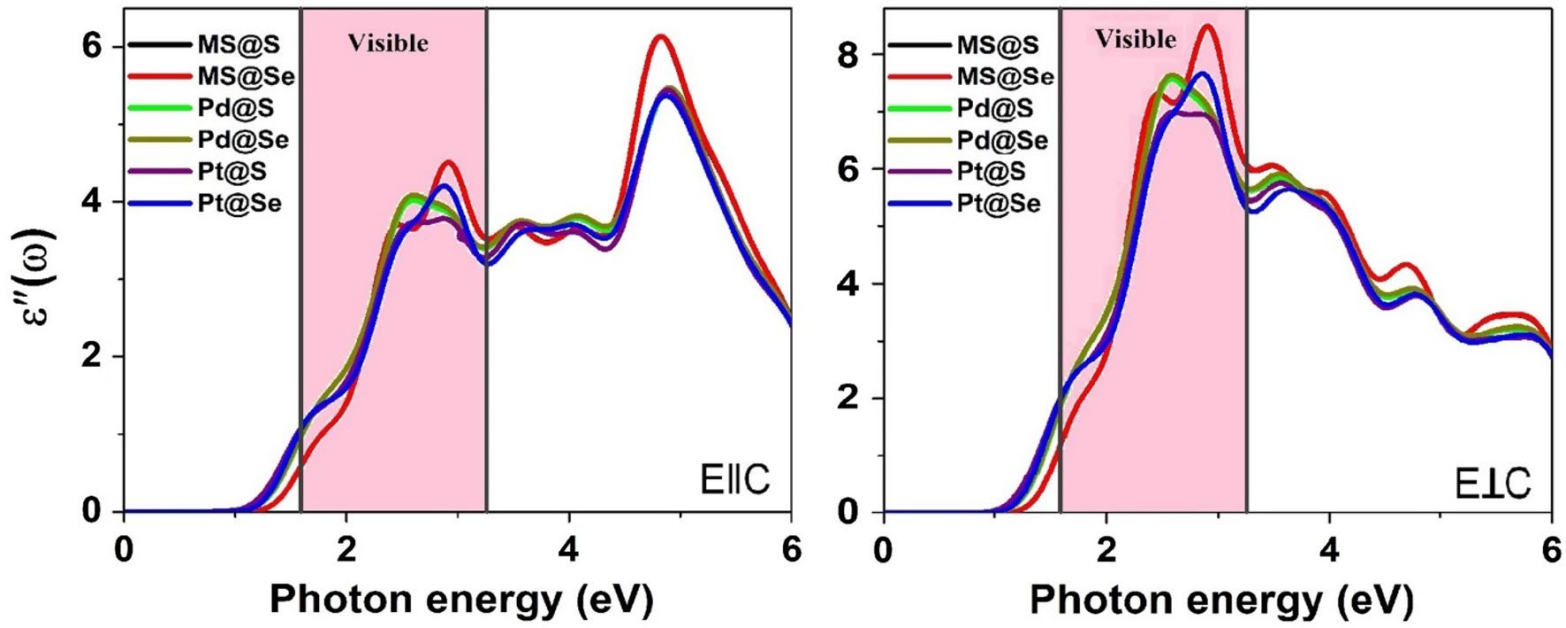
The imaginary part of dielectric function for pristine and Pd/Pt decorated MoSSe monolayers after CH_4_ adsorption. Here, MS@S and MS@Se represent adsorption on S and Se sites of MoSSe monolayer, respectively.

The optical gas sensor has high sensitivity and selectivity in contrast to conductivity-based gas sensors^46,47^. Considering this aspect, we have focused on imaginary part of dielectric function along both direction parallel and perpendicular to vector axis. This response reveals strong anisotropy in UV–VIS region as displayed in Fig. 5 and S5 where dielectric response ε″(ω) is higher toward parallel direction compared to same along perpendicular direction. The intense peak for pristine MoSSe monolayer at 4.82 eV in near UV region toward parallel direction exhibits red shift with maximum peak at 2.89 eV in visible region along out-of-plane direction. Also, it is clearly seen that behavior of ε″(ω) in the proximity of pristine MoSSe monolayer vary slightly after CH_4_ adsorption. Besides, overall behavior of ε″(ω) in the vicinity of Pd/Pt decorated MoSSe monolayers is identical for S and Se sites, individually before adsorption. In contrast, dielectric response of Pd/Pt decorated MoSSe monolayers modulated significantly within energy range of about 2.2–3 eV in visible region after activation of CH_4_ molecule. To this alteration, ε″(ω) of same monolayer exhibits blue shift in UV–VIS region under the effect of CH_4_. Moreover, a new peak is introduced at 2.59 eV when Pd decorated monolayers exposed to CH_4_. Finally, response of ε″(ω) increases and decreases for Pd and Pt decorated MoSSe monolayers, respectively in visible region after CH_4_ adsorption. This variation indicates sensitivity toward methane in comparison with that of pristine which may detect CH_4_ gas easily in visible region.

## Conclusions

We have effectively studied impact of Pd/Pt modification on MoSSe monolayer to enhance adsorption capacity of CH_4_ and examined sensing parameters using DFT. Firstly, we explored structural and electronic properties of pristine MoSSe monolayer and observed variation for the same in the presence of Pd/Pt. This result of electronic band structure for pristine monolayer confirms semiconducting character with direct band gap of 1.52 eV. Similarly, Pd/Pt decorated monolayers exhibit semiconducting nature with decreased band gap. Further, to CH_4_ adsorption, we considered both active sites S and Se of all monolayers. The adsorption energy significantly increases for CH_4_ adsorption in the presence of Pd/Pt adatom, indicating higher binding strength of CH_4_ toward Pd/Pt decorated MoSSe monolayers compared to pristine monolayer. Though, we found maximum adsorption energy as − 0.674 and − 0.636 eV for adsorption on Se site of Pd/Pt decorated MoSSe monolayers, overall sensing parameters reveal both sites, S and Se are favorable for CH_4_ adsorption. The results clearly indicate that electronic properties of pristine MoSSe monolayer are not affected strongly by CH_4_. However, Pd/Pt decorated MoSSe monolayer when exposed to CH_4_, shows transformation to direct band gap with marginal alteration. Moreover, significant variation in optical dielectric response is noticed in the visible region after activation of CH_4_ on to Pd/Pt decorated MoSSe monolayer. This result emphasis better sensitivity of Pd/Pt decorated MoSSe monolayers toward CH_4_ which points out easy detection of CH_4_ in the visible region. Generally, this conceptual sensing study of methane promotes Pd/Pt decorated MoSSe monolayers as good nano-sensing devices.

### Supplementary Information

Supplementary Figures.

## Data Availability

The datasets generated and/or analyzed during the current study are not publicly available due to privacy or other restrictions. However, it may be made available by the corresponding author on reasonable request.
