# Supplementary material for: Effect of Pd/Pt decoration on MoSSe monolayer for CH4 signature through surface adsorption mechanism
Source: Sci Rep. 2023 Dec 12;13:22062. doi: 10.1038/s41598-023-49028-x (PMC10716147; doi:10.1038/s41598-023-49028-x)
Supplement: Supplementary file 1 — Supplementary Figures. [file 41598_2023_49028_MOESM1_ESM.docx]

**SUPPLEMENTARY MATERIAL**

Effect of Pd/Pt decoration on MoSSe monolayer for CH_4_ signature through surface adsorption mechanism

# Bindiya Babariya^1^, Sanjeev K. Gupta^2^ and P. N. Gajjar^1^

1Department of Physics, University of School of Sciences, Gujarat University, Ahmedabad-380 009, Gujarat, India

2Computational Materials and Nanoscience Group, Department of Physics, St. Xavier's College, Ahmedabad 380009, India

*pngajjar@gujaratuniversity.ac.in, pngajjar@rediffmail.com; sanjeev.gupta@sxca.edu.in


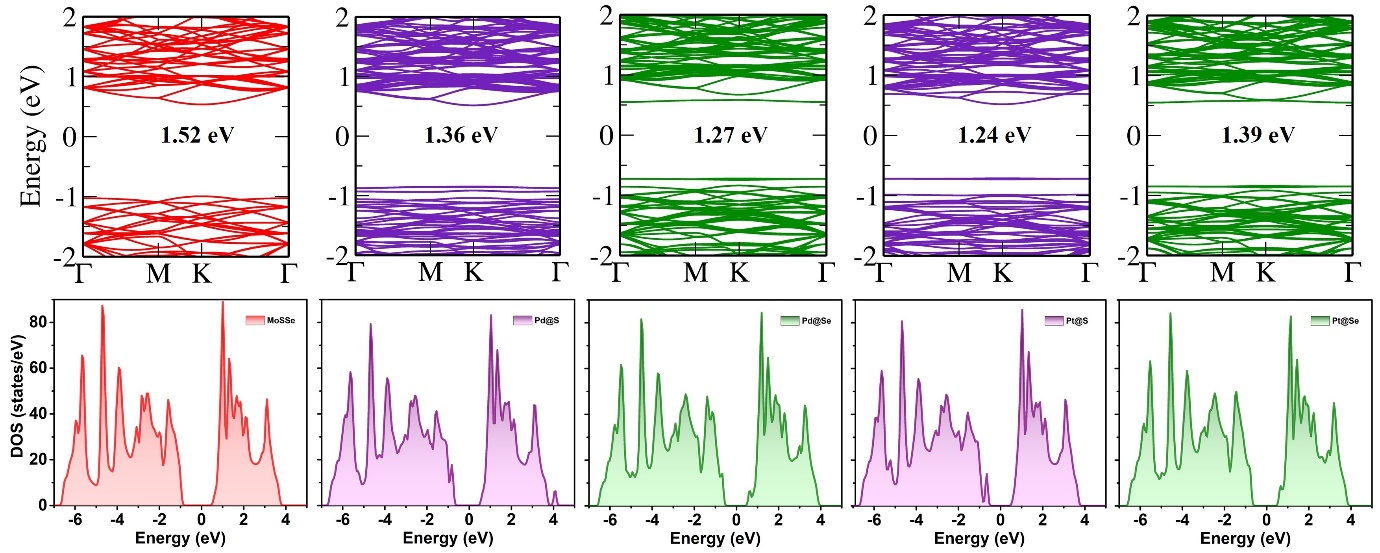


Figure S1 The electronic band structure and corresponding DOS (using SOC) for pristine and Pd/Pt doped MoSSe monolayers. The Fermi level is set to zero.


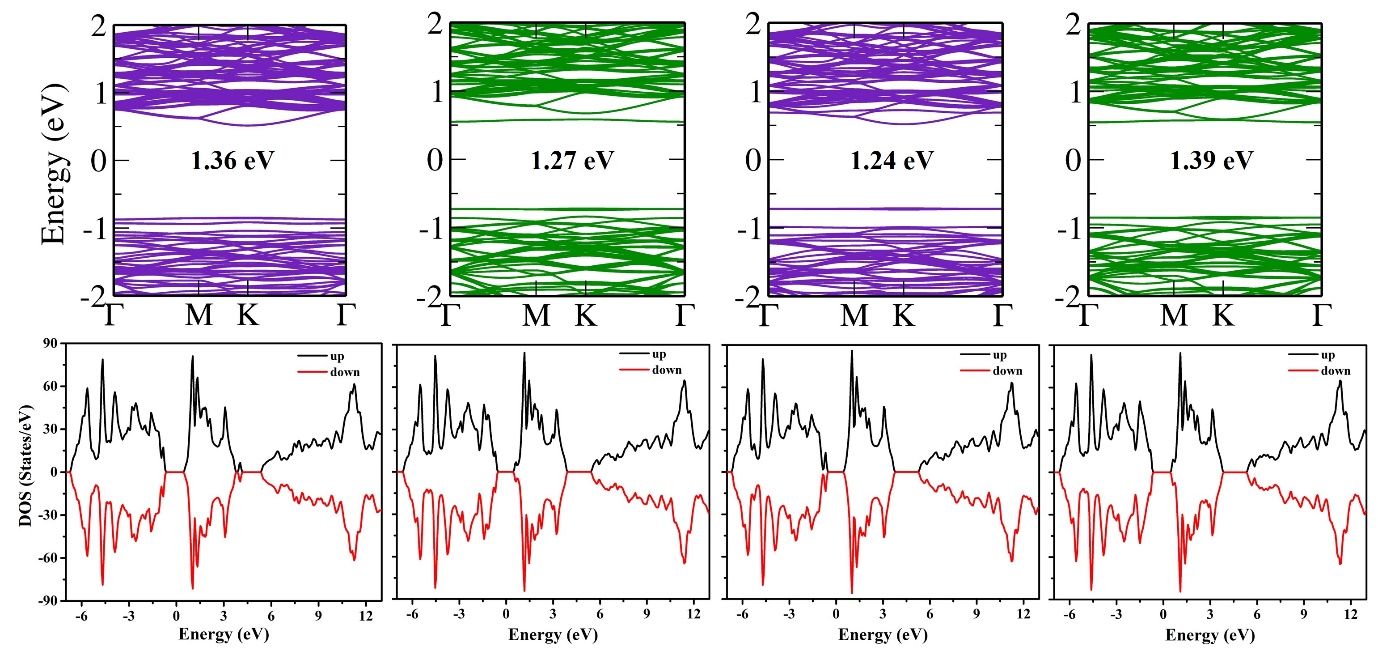


Figure S2 The electronic band structure and corresponding DOS (using Spin Polarizations) for pristine and Pd/Pt doped MoSSe monolayers. The Fermi level is set to zero.


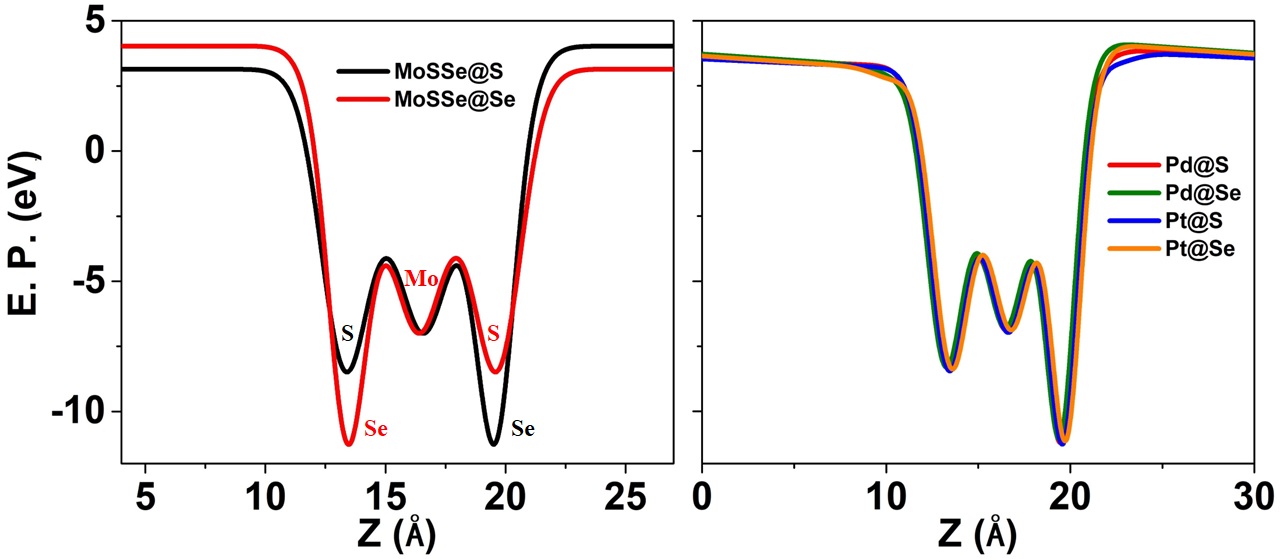


Figure S3 Electrostatic potential for pristine and Pd/Pt doped MoSSe monolayers before adsorption.


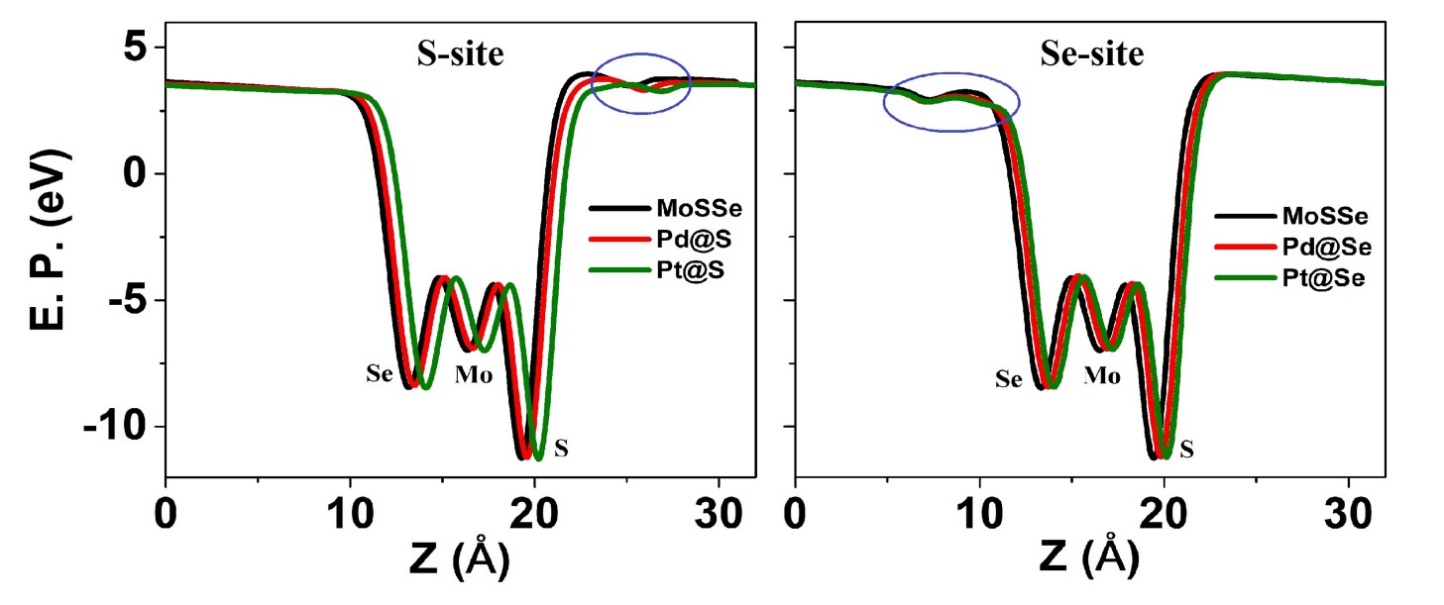


Figure S4 Electrostatic potential for pristine and Pd/Pt doped MoSSe monolayers after CH_4_ adsorption


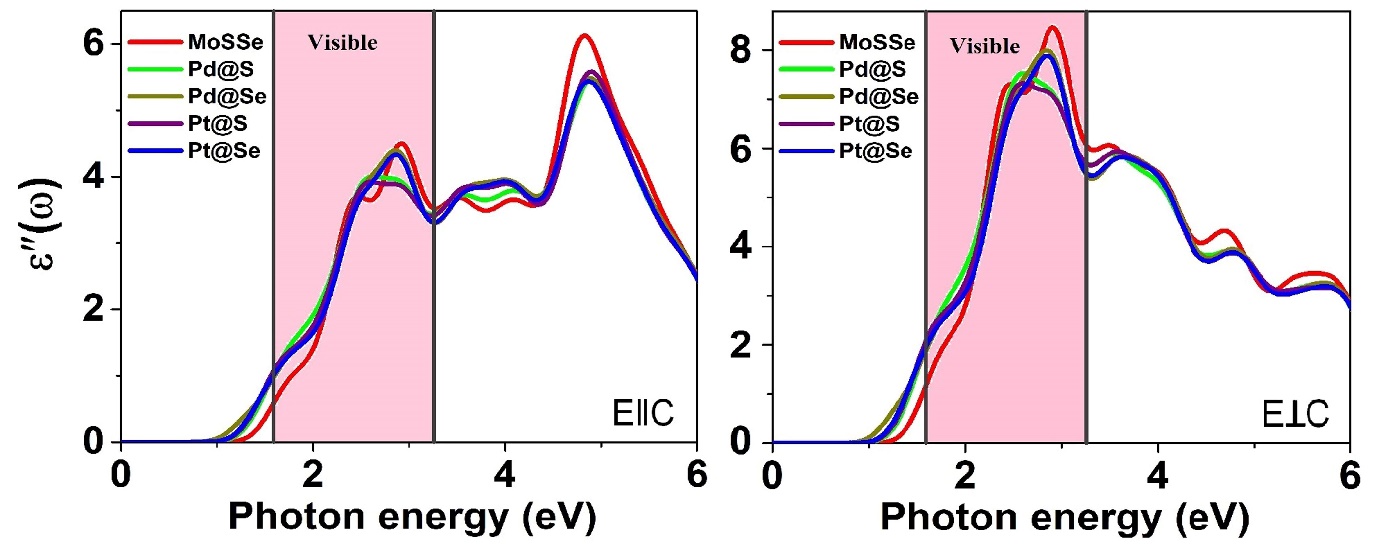


Figure S5 The imaginary part of dielectric function for pristine and Pd/Pt decorated MoSSe monolayer before CH_4_ adsorption
